# Drug-susceptible tuberculosis treatment outcomes and its associated factors among inmates in prison settings in Bauchi State, Nigeria, 2014–2018

**DOI:** 10.1371/journal.pone.0270819

**Published:** 2022-07-05

**Authors:** Peter Okpeh Amede, Elizabeth Adedire, Aishat Usman, Celestine Attah Ameh, Faruk Saleh Umar, Chukwuma David Umeokonkwo, Muhammad Shakir Balogun

**Affiliations:** 1 Nigeria Field Epidemiology and Laboratory Training Program, Abuja, Federal Capital Territory, Nigeria; 2 African Field Epidemiology Network- Nigeria, Abuja, Federal Capital Territory, Nigeria; 3 Nigerian Correctional Service, Bauchi State Command, Abuja, Federal Capital Territory, Nigeria; 4 Department of Community Medicine, Alex Ekwueme Federal University Teaching Hospital, Abakaliki, Ebonyi State, Nigeria; The University of Georgia, UNITED STATES

## Abstract

Tuberculosis (TB) is a contagious disease and its transmissibility is increased in congregate settings. TB incidence rates are five-to-fifty times higher among inmates in prison settings than the general population which has a direct impact on the outcome of TB treatment. There is paucity of information on TB treatment outcomes and its associated factors in Nigerian prison settings. We therefore assessed TB treatment outcomes among inmates in prison settings in Bauchi State, Nigeria. We conducted a retrospective data analysis of inmates with TB in the five-main prison settings in Bauchi State. We extracted socio-demographic, clinical and treatment outcome characteristics from TB treatment register of inmates treated for TB between January 2014 and December 2018, using a checklist. We calculated the TB treatment success rate (TSR) and explored the relationship between the TSR and socio-demographic and clinical characteristics. Related variables were modelled in multiple logistic regression to identify factors associated with TSR at 5% level of significance. All 216 inmates were male with mean (SD) age of 37.6±11.4 years. Seventy-six (35.2%) were cured, 61 (28.2%) completed treatment, 65 (30.1%) were transferred-out without evaluation and 14 (6.5%) died. Overall TSR was 72.9%. Factors associated with successful-treatment-outcome were age, weight, imprisonment duration and HIV status. The results indicate that inmates who are 20–29 years are at least ten times more likely to be successful (aOR = 10.5; 95%CI: 3.2–35.1) than inmates who are 55 years or older. Inmates who are 30–39 years are about four times more likely to be successful than inmates who are 55 years or older (aOR = 4.2; 95% CI: 1.3–13.1). In general, the younger an inmate, the more successful he is. Inmates with pretreatment-weight; 55kg or more are 13 times more likely to be successful (aOR = 13.3; 95%CI: 6.0–29.6) than inmates with weight below 55kg. Inmates who were imprisoned for 2 years or less are about three times more likely to be successful (aOR = 2.6; 95%CI: 1.3–5.4) than inmates who were imprisoned for more than 2 years and HIV negative inmates were three times more likely to succeed (aOR = 3.3; 95%CI:1.4–7.8) than inmates who were HIV positive. We recommended that to improve TB treatment outcome among inmates; age, duration-of-imprisonment, weight and TB/HIV co-infection should be the major consideration during pretreatment, psychological and nutritional counselling and a tracking-system be developed by the authority to follow-up inmates transferred-out to other health facilities to ensure they complete the treatment and outcomes evaluated.

## Introduction

Tuberculosis (TB) is a preventable and curable disease with effective drugs, despite this it remain a major killer with over 4,500 dying daily from the infection globally [1, 2]. In 2020, TB was reported worldwide as the 13^th^ leading cause of death; with 10 million cases and 1.5 million deaths attributed to the disease including 214,000 deaths from TB among those co-infected with HIV [3]. The high global prevalence of TB is driven by HIV infection, lack of TB diagnostic laboratories, poverty and weak healthcare systems [4]. Nigeria ranked 6^th^ in the world and 1^st^ in Africa among the 30 countries with the highest burden of TB, in 2020 [3]. In Nigeria, TB deaths including HIV/TB co-infection was 155,000 in 2017; which was second only to India [5].

Tuberculosis transmissibility potential is increased in congregate settings such as prisons due to a high prevalence of HIV infection among persons in prison, overcrowding, poor nutrition, poor hygiene, prolonged indoor confinement without adequate ventilation and limited access to healthcare [6–8]. Globally, TB burden among persons in prison is 5–50 times higher than among the general population and is estimated to be the leading cause of death among persons in prison [9]. A case of active Pulmonary TB (PTB) can infect 10–15 persons over the course of a year, this might be higher among prison population due to overcrowding and prolonged close contacts [10, 11]. In sub-Saharan African prisons, TB remains one of the fastest growing infectious diseases [11–13]. Overcrowding in Nigerian prison settings has been on a steady rise; the total population in 2018 was 73,631 up from 44,450 in 2000 and 57,313 in 2015 without a corresponding expansion of the capacity of the prison settings [14].

The goal of TB treatment is to cure those with TB disease, prevent deaths from the disease and stop transmission of tubercle bacilli from individual with active PTB to susceptible individuals [15]. In 2017, TB treatment saved 53 million people globally, including HIV positive TB cases and 11 million individuals were saved in Africa [16]. TB Treatment outcome is an indicator for evaluating TB control programmes and it is influenced by socio-demographic characteristics, socio-economic factors (such as poverty, housing), nutrition, HIV co-infection, Multi-Drugs Resistant TB (MDR-TB), and strategies for TB management including Directly Observed Treatment short (DOTs) course [2, 17, 18].

Tuberculosis treatment success is the sum of cured and treatment completed, and unsuccessful TB treatment outcome is the sum of treatment failure, lost to follow-up, transferred out without evaluation and died. Treatment Success Rate (TSR) is the percentage of all new smear-positive TB cases in a given year that successfully completed treatment with bacteriological evidence (cured) or without bacteriological evidence (treatment completed) of success among all who commenced the treatment. The numerator is TB cases that successfully completed treatment and the denominator are the new cases. World Health Organization (WHO) set ≥ 90% as the global target for TSR to eliminate TB as a global public health concern and a cure rate of ≥85% [19]. The TB TSR globally among all new TB cases was 83% in 2017 and the corresponding TSR for sub-Saharan Africa was 76% [17, 19]. In 2015, TB TSR in Nigeria was 84% below the WHO target, and ranked 84^th^ in the global rating and 23^rd^ in Africa [20].

TB TSR among persons in prison in the European region in 2015 was 59.7%, likewise a study in 2017 among 162 persons in prison with TB in Ethiopia, reported TSR of 63.62% [20, 21]. No available data for TB TSR among prison population in Nigeria.

The prison population is a dynamic and unstable one and this might impact negatively on TB treatment outcomes. Poorly treated TB cases in the prison could compound TB burden with-in the prison and increase the risk of MDR-TB. Studies have reported trends in TB treatment outcomes among the general population in Nigeria and for different states within the country [16, 22, 23]; however, there is limited information about factors that influence TB treatment outcome among inmates in prison settings in Nigeria, despite several factors have been known to influence standard TB treatment outcomes among the general population. We therefore conducted this study to assess TB treatment outcomes and its associated factors among inmates in prison settings in Bauchi State, Nigeria to audit the effectiveness of the TB control programme in the state prison settings and the findings will help to develop specific interventions to correct identified gaps.

## Material and methods

### Study setting, diagnostic criteria and treatment regimen

The study was conducted in all the five main prison settings (Bauchi, Azare, Ningi, Misau and Jama’are) in Bauchi State. The facilities housed both male and female inmates and lock-up above its maximum capacity and the holding cells are usually overcrowded. The maximum capacity of Bauchi prison is 500, Azare 320, Misau 120, Ningi 110 and Jama’are 151. At the time of the study in February 2019, the total inmates’ population in the 5 prison settings was 2106 out of this figure, eleven (0.5%) were females. Bauchi prison had 1006 inmates (male = 997; female = 9), Azare 501 (male = 500; female = 1), Misau 204 (all male), Ningi 159 (male = 158; female = 1) and Jama’are 236 (all male). The prison settings have clinics with various cadres of healthcare workers (Nurses, community health officers, pharmacy assistants, dental assistant, community health extension workers, laboratory technologists, radiographers and one medical doctor that over sees the prison settings in the state) that provides mainly curative services for inmates, staff and staff relations. These prison clinics were poorly equipped with no Gene Xpert, sputum microscopy or drug susceptibility testing services. TB diagnosis in these facilities relied mainly on referral of inmates with presumptive TB to the DOTs centres of the public health facilities outside the prison settings. Bauchi prison refer to State specialist hospital, Bauchi; Azare to federal medical centre, Azare; Ningi, Jama’are and Misau refer to Ningi, Jama’are and Misau general hospital respectively. These DOTs centres are supported by the National Tuberculosis and Leprosy Control Programme (NTLCP), Bauchi state coordinating unit. Laboratory diagnosis, treatment procedures, drug regimens, and follow-up assessment are provided according to the National TB, Leprosy and Burili ulcer management and control guidelines 2014 [24]. Presumptive TB case is an inmate with cough of ≥ 2 weeks with at least one of the following symptoms; fever, night sweats, shortness of breath, chest pain, haemoptysis and or unintentional weight loss, and for HIV positive inmates any duration of cough is presumed to be TB. Inmates diagnosed of TB by the public health facilities are placed on Anti-TB drugs for the full course by the public health facility that confirmed the diagnosis but continued the treatment in the prison under the supervision of the TB desk officers, who also kept the TB treatment cards and registers. Inmates diagnosed with TB are kept in isolation cells within the facility during the intensive phase of treatment and the drugs are administered through the DOTs strategy. The treatment is based on new (a patient who has never had treatment for TB, or have taken anti-TB drugs for less than 4weeks) or retreatment/previously treated (are patients who have received 4 weeks or more of anti-TB drugs in the past) TB cases; standard six month treatment regimen (Regimen 1) for all form of TB (PTB and EPTB cases–both new and previously treated) and Regimen 2 for TB meningitis and Osteo-articular TB cases; that receive twelve month treatment regimen (2 months of intensive and 10 months of continuation phase). Regimen 1 (six months treatment regimen) consist of two months of intensive phase of combination of four drugs (Rifampicin, Isoniazid, Pyrazinamide and Ethambutol) and four months of continuation phase of two drugs (Rifampicin and Isoniazid). Regimen 2 (twelve months treatment regimen) consist of two months of intensive phase of combination of four drugs (Rifampicin, Isoniazid, Pyrazinamide and Ethambutol) and ten months of continuation phase of two drugs (Rifampicin and Isoniazid). The dosage of the drugs depends on the patient’s pre-treatment weight. Monitoring of TB treatment is an essential part of TB case management. Follow-up bacteriological test is done for all inmates with TB who had sputum examined at diagnosis. It involves examination of one early morning sputum sample for AFB at different points during treatment. Weight monitoring is part of clinical assessment which is done at the end of the second, fifth and at the end of the last month of treatment. All presumptive TB cases are counselled and tested for HIV using determine (rapid HIV test kit), positive cases were confirmed with Stat-Pak (rapid HIV diagnostic kits), while Uni-Gold recombinant assay is used as tie-breaker for discordant results.

### Study design and population

We conducted a retrospective prison-based study of TB patient’s records from January 2014 to December 2018 in all five main prison settings in Bauchi State. All 216 inmates treated for TB with Anti-TB drugs from January 2014 to December 2018 were included.

### Data tool and collection

The data source was the TB treatment register and patient health cards in the five selected prison settings. Data were extracted using a structured checklist. The checklist collected information on age, pretreatment weight, duration of imprisonment at the time of diagnosis, TB class (Smear Positive Pulmonary Tuberculosis [SPPTB], Smear Negative Pulmonary Tuberculosis [SNPTB], Extra-Pulmonary Tuberculosis [EPTB]), treatment category (New, Unknown and Re-treatment), HIV status (HIV-positive TB patient and HIV-negative TB patient), treatment outcomes (cured, treatment completed, failure, transferred out without evaluation and died), from the TB treatment registers in the five studied prison settings by five trained healthcare workers (HCWs). The five HCWs (one from each facility) were trained for a day on how to collect the data and use the checklist. The principal researcher daily reviewed the filled format and strictly supervised the trained research assistants. Data were collected over a period of five weeks. Each facility was assigned a week; this enable the researcher to supervised the process of data abstraction.

### Measurement

The dependent variable (treatment outcome) was dichotomized as successful (cured and treatment completed) and unsuccessful (treatment failure, transferred out without evaluation and died) and the independent variables were socio-demographic characteristics (age, pre-treatment weight, duration of imprisonment) and clinical characteristics (HIV status, TB class and treatment history). Sensitivity analysis: The process of changing or excluding the values of some parameters, variables, and/or model structure to examine the robustness of the results.

The following operational definitions were adopted from WHO for drug-susceptible TB [25].

Treatment Success Rates (TSR): Proportion of smear-positive TB cases registered under DOTs in a given year that successfully completed treatment, whether with (cured) or without (treatment completed) bacteriologic evidence of success.

Cured: A PTB case with bacteriologically confirmed TB at the beginning of treatment who was smear or culture negative in the last month of treatment and on at least one previous occasion.

Treatment completed: A TB case who completed treatment without evidence of failure but without records to show that sputum smear or culture results in the last month of treatment and on at least one previous occasion were negative, either because tests were not done, or results were unavailable.

Treatment failure: A TB case whose sputum smear or culture is positive at month 5 or later during treatment.

Lost to follow-up: A TB case who did not start treatment or whose treatment was interrupted for two consecutive months or more.

Not evaluated: A TB case for whom no treatment outcome is assigned. This includes cases transferred out to another treatment unit as well as cases for whom the treatment outcome is unknown to the reporting unit.

Died: A TB case who dies for any reason during treatment.

New TB patient: A TB case who has not previously been treated or treated for less than a month for TB and is now diagnosed and has started the current treatment.

Retreatment/Previously treated: A TB case who was previously treated for TB and was declared cured and now diagnosed and started the current treatment. Called relapse in the past.

### Data analysis

Abstracted data were checked for its completeness, correctness and analyzed using Epi-info software version 7.2 (CDC, Atlanta, USA). Descriptive statistics was used to generate summary frequencies, percentages, means and standard deviation. Sensitivity analysis was done by excluding TB cases that were not evaluated to test its effect on the treatment success rate. Bivariate analysis was performed to measure association between treatment outcome and independent variables. Covariates with p-value of ≤ 0.05 in the bivariate analysis were included in multiple logistic regression model to identify factors associated with treatment outcomes at 5% significant level.

### Ethical consideration

Ethical approval was obtained from the Bauchi State Health Research Ethics Committee with protocol approval number: NREC /03/11/19B/2021/19. Permission was sought and obtained from the Controller of Correctional Service, Bauchi State command, where the aim and objectives of the study were explained. The information obtained was made anonymous and de-identified prior to analysis to ensure confidentiality.

## Results

Although there were eleven females incarcerated during the study period, all the 216 TB patients registered during the study period were males. The mean age (SD) was 37.6 (±11.4) years with 132 (61.1%) less than 40 years. The mean (SD) weight was 55.2 (±9.8kg) with 111 (51.4%) having weight ≥ 55kg, 97 (44.9%) incarcerated in Bauchi prison, the median (Inter Quartile Range) duration of imprisonment was 21 months (14–28 months) and 134 (62.0%) were imprisoned for less than 2 years. Most were new cases 152 (70.4%), majority 188 (87.0%) had SPPTB, and about a quarter 46 (21.3%) were co-infected with HIV as shown in Tables 1 and 2.

**Table 1:** Socio-demographic characteristics of TB patients treated in five main prison settings in Bauchi State from January 2014 to December 2018.

Variable	Number (N = 216)	Percentage
Age (years)		
20–29	64	29.6
30–39	68	31.5
40–49	50	23.2
≥50	34	15.7
Weight (kg)		
≥55	111	51.4
<55	105	48.6
Facility formation		
Azare	40	18.5
Bauchi	97	44.9
Jama’are	30	13.9
Misau	24	11.1
Ningi	25	11.6
Year of enrolment		
2014	54	25.0
2015	64	29.6
2016	42	19.5
2017	34	15.7
2018	22	10.2
Duration of imprisonment		
≤2 years	134	62.0
>2 years	82	38.0

**Table 2:** Clinical characteristics of TB patients treated in five main prison settings in Bauchi State from January 2014 to December 2018.

Variable	Number (N-216)	Percentage
Treatment history		
New	152	70.4
Retreatment	21	9.7
Unknown	43	19.9
HIV status		
Positive	46	21.3
Negative	170	78.7
TB class		
SPPTB**	188	87.0
SNPTB^+^	6	2.8
EPTB^⸸^	22	10.2

**Smear Positive Pulmonary Tuberculosis

+Smear Negative Pulmonary Tuberculosis

⸸Extra Pulmonary Tuberculosis

**Fig 1:**
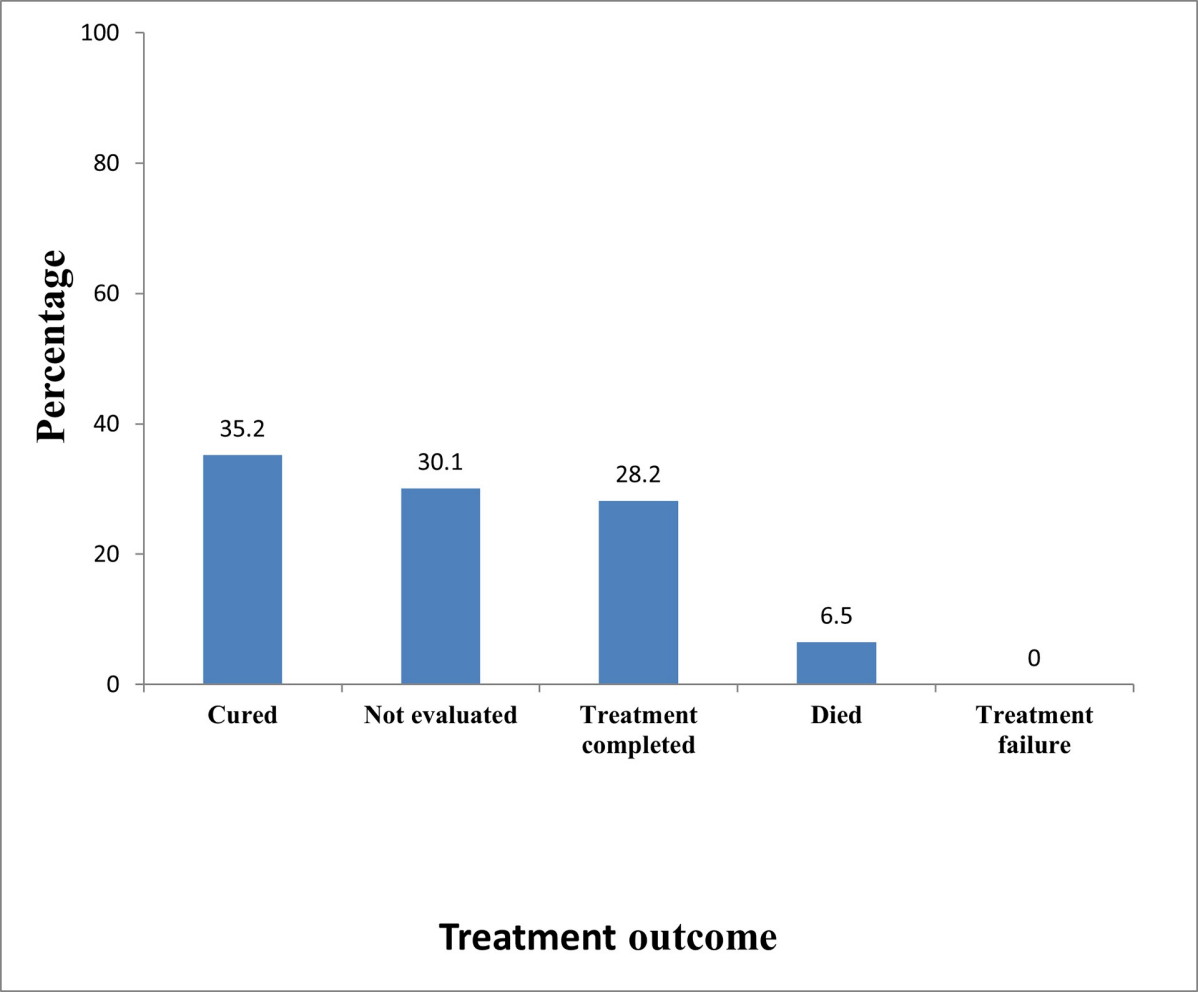
Treatment outcome of TB cases in the five main prison settings in Bauchi State from January 2014 to December 2018.

Seventy-six (35.2%) were cured while 61 (28.2%) had completed treatment, with no treatment failure recorded during the study period. Sixty-five (30.1%) were transferred out without evaluation and 14 (6.5%) patients died as seen in Fig 1.

**Fig 2:**
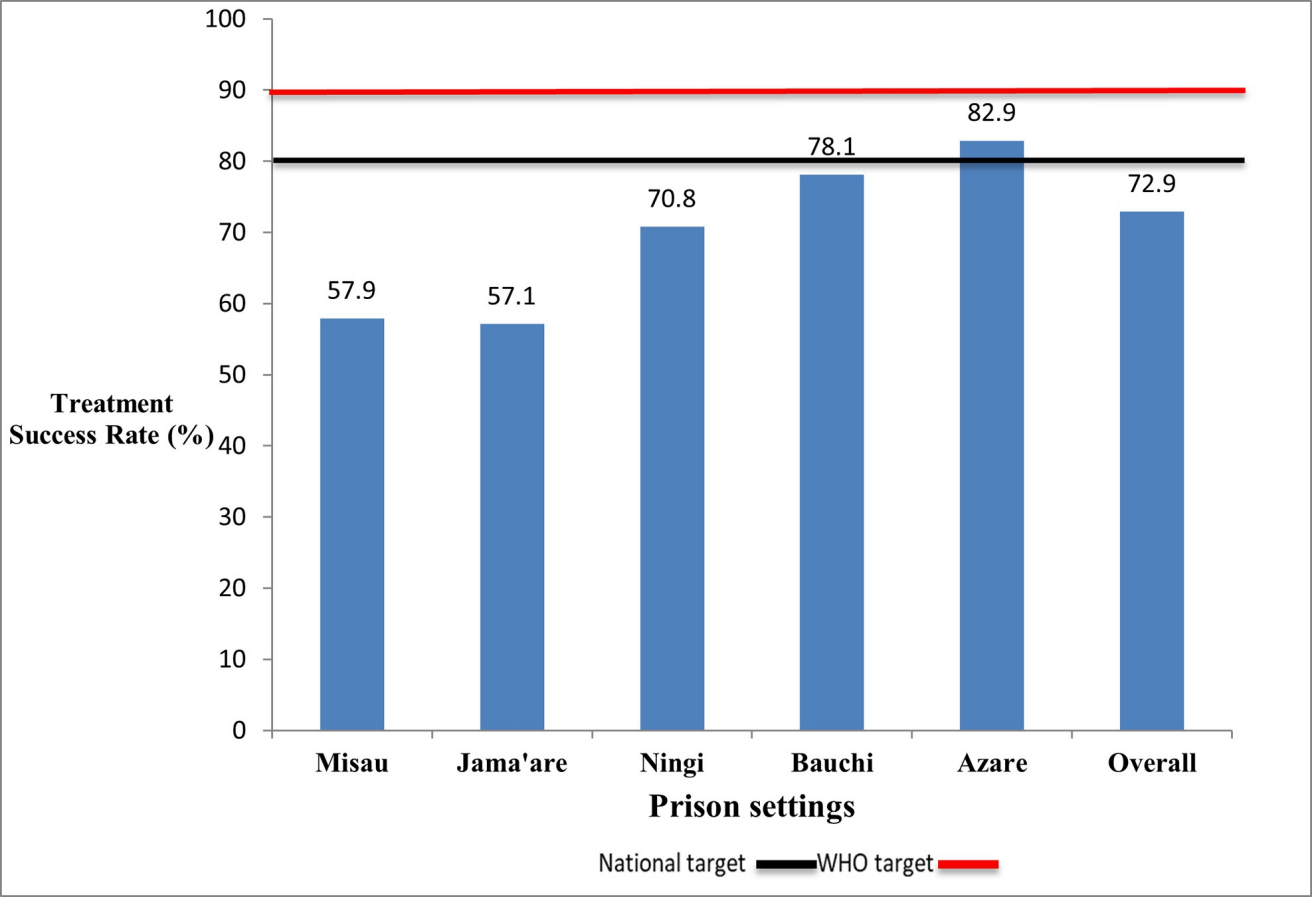
Treatment success rates of TB patients in the five main prison settings in Bauchi State from January 2014 to December 2018.

The overall TSR was 72.9%; and 95.1% when sensitivity analysis was done by excluding TB cases whose treatment outcomes were not evaluated. The highest TSR was observed in Azare prison (82.9%) and the lowest in Jama’are prison (57.1%), Bauchi prison (78.1%), Ningi prison (70.8%) and Misau prison (57.9%), over the five year-period. The difference in the treatment outcome across the five prison was not statistically significant p = 0.1581 (Fig 2).

**Fig 3:**
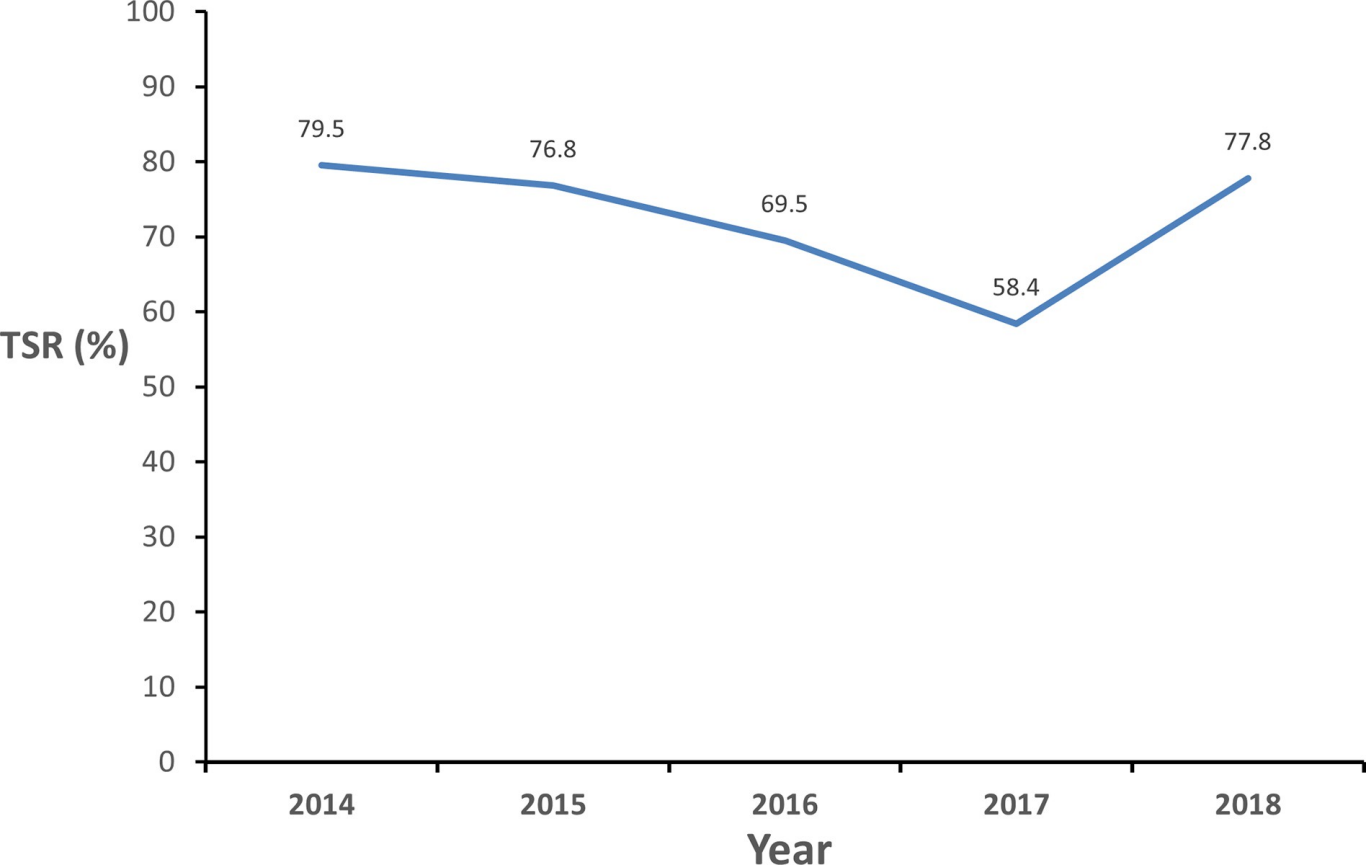
Trends of treatment success rates of TB patients in five main prison settings in Bauchi State from January 2014 to December 2018.

The trend in TSR of all TB patients decreased from (79.5%) in 2014 to (76.8%) in 2015, to (69.5%) in 2016, further down to (58.4%) in 2017 and rose to (77.8%) in 2018, as depicted in Fig 3. TSR was higher in HIV negative patients (77.1%) compared to HIV positive patients (57.1%) p< 0.001. TSR was higher among age group 20–29 years (89.5%), compared to 30–39 years (83.1%), 40–49 years (56.8%), lowest among age group ≥50years (42.9%); p = 0.0002 and in those with weight ≥55kg (99.0%) compared to <55kg (43,2%); p<0.001.

Factors associated with successful-treatment-outcome were age, weight, imprisonment duration and HIV status. The results indicate that inmates who are 20–29 years are at least ten times more likely to be successful (aOR = 10.5; 95%CI: 3.2–35.1) than inmates who are 55 years or older. Inmates who are 30–39 years are about four times more likely to be successful than inmates who are 55 years or older (aOR = 4.2; 95% CI: 1.3–13.1). In general, the younger an inmate, the more successful he/she is. Inmates with pretreatment-weight; are 13 times more likely to be successful (aOR = 13.3; 95%CI: 6.0–29.6) than inmates with weight below 55kg. Inmates who were imprisoned for 2 years or less are about three times more likely to be successful (aOR = 2.6; 95%CI: 1.3–5.4) than inmates who were imprisoned for more than 2 years and HIV negative inmates were three times more likely to succeed (aOR = 3.3; 95%CI:1.4–7.8) than inmates who were HIV positive as shown in Table 3.

**Table 3:** Factors associated with successful treatment outcome of tuberculosis in five main prison settings in Bauchi State from January 2014 to December 2018 according to multivariate analysis.

Variables	Successful n (%)	Unsuccessful n (%)	aOR
Age (years)			
20–29	51 (79.7)	13 (20.3)	**10.5 (3.2–35.1)** ^*^
30–39	49 (72.1)	19 (27.9)	**4.2 (1.3–13.1)** ^*^
40–49	25 (50.0)	25 (50.0)	**0.3 (0.1–0.7)** ^*^
≥50	11 (32.3)	23 (67.7)	1
Duration of imprisonment			
≤2 years	99 (72.8)	35 (43.8)	**2.6 (1.3–5.4)** ^*^
>2	37 (27.2)	45 (56.2)	1
HIV status			
Negative	117 (86.0)	47 (66.3)	**3.3 (1.4–7.8)** ^*^
Positive	19 (14.0)	27 (33.7)	1
Weight (kg)			
≥ 50	98 (72.1)	13 (16.2)	**13.3(6.0–29.6)** *
< 50	38 (27.9)	67 (83.8)	1

* Statistically significant at 5% level; aOR-Adjusted Odds Ratio

## Discussion

This paper describes TB treatment outcome and its associated factors among male inmates in prison settings in Bauchi State. We found a high treatment success rate among the inmates compared to findings in other prison settings but below the national and international minimum recommended rate. The high TSR could be due to the dedication of trained TB desk officers in all the prisons with administration of the drugs under strict supervision and the inmates are readily accessible to the desk officers. This may also be because of the confined nature of the environment which made it difficult for the patient to be lost to follow up and hence poor adherence. This could also be due to the fact that persons in prison do not need to bother about transportation cost to treatment centres as that is one of the predictors to non-adherence to treatment among TB patients in the general population [26]. The TB TSR in this study indicates that the TB control programme in prison settings in Bauchi State is effective especially for inmates who completed their treatment in the facilities. The TSR in this study is higher than that recorded among persons in prison in European region and in Debribirhan prison Ethiopia but lower compared to that found among inmates in Malawi prisons and Northern Ethiopian prisons [20, 21, 27, 28]. This finding is due to the fact that about a third of the TB patients were transferred out of the treatment facilities during treatment without follow-up to evaluate the treatment outcomes. The public health implication is that some of these TB patients might not adhere to the treatment regimen or complete the treatment duration which could lead to prolong morbidity, continuous transmission of MTB within the host community, development of drugs resistance MTB, Multidrug-Resistant TB (MDR-TB), Extensively Drug Resistance TB (XDR-TB) and increase TB related deaths [29, 30]. This can reverse the gains in the TB control programme and the achievements made towards the WHO end the global TB epidemic by 2030.

One of the key determinants of an effective TB control programme is access to TB treatment. Tuberculosis treatment is free for inmates in prison settings in Nigeria as it is for the general population. A sound understanding of the factors associated with TB treatment outcomes can lead to appropriate interventions to improve the outcome. The high rate of transferred out without evaluation negatively impacted on the TSR in this study. The TSR was 95.1% after excluding TB cases that were not evaluated, indicating the negative impact of this on the TSR in this study. This is similar to other studies where major cause of unsuccessful treatment outcome was due to transferred-out cases without evaluation [31, 32].

Tuberculosis TSR provides a useful indicator for assessing the effectiveness of TB control programme. A low rate suggests that infectious patients may not be receiving adequate treatment and stand the risk of developing drug resistant TB and could serve as a potential reservoir for the transmission of MDR-TB. The overall TSR for this study is lower than the WHO set target and the Nigeria national average but similar to the rate among persons in prison in Ethiopia and higher than the rate among inmates in Uganda [20, 21, 33]. The lower TSR in this study compared to the WHO target and the Nigeria national average results from the large number of TB patients transferred-out without evaluation of their treatment outcomes, and the significant cases of TB/HIV co-infection which is a predictor for poor TB treatment outcome in this study. A high proportion of TB cases not evaluated demonstrate poor tracking system due to weaknesses in Monitoring and Evaluation and lack of supervision.

The cure rate in this study is higher than the studies conducted in Northern Ethiopian prisons and in North Shoa, Ethiopia but lower than that recorded in El-Salvador prison [21, 27, 34]. The low cure rate in this study compared to the El-Salvador study could be attributed to the lower sample size in this study and the high rates of transferred-out, since there was no system to track their progress, the final treatment outcomes of these patients were not evaluated.

All the TB patients studied were males, this is similar to the study among persons in prison in North Shoa Ethiopia and Zomba Malawi [21, 28]. The prison population is predominantly male and most prisons are male only institutions, including prisons staff. Worldwide, females makes-up just 7% of prison population, and is much lower in African countries including Nigeria [35]. Female inmates constitute 2% of the total prison population in Nigeria [35]. Over three-fifth of the patients studied were in the age group <40 years, this is similar to other findings among persons in prison with TB in North Shoa, Ethiopia but lower than that found among persons in prison in North West province, South Africa and in the general population in Western Ethiopia [9, 21, 36]. This could be so because of high mobility, high criminal activities and imprisonment among this age group.

We found out that age, duration of imprisonment, weight and HIV status were associated factors for successful TB treatment outcome in this study. The odds of successful treatment outcome decrease with advancing age, this is comparable with the study in Zimbabwe on age-stratified tuberculosis treatment outcomes and other studies, where the elderly had a poorer treatment outcome compared to the younger patients [37–40], this might be due to better immune response among the younger age group and probably other associated comorbidities among the older age group. Duration of incarceration was significantly associated with successful treatment outcome; this is similar to the finding among inmates in Ethiopia prisons [27]. This finding could be attributed to poor adherence to treatment protocol due to difficulty of prison life and mental stress associated with prolonged incarceration. The odds of successful treatment outcome increase with heavier body weight. Similarly, a study in Ethiopia revealed that pretreatment weight category of 55.0–70.9kg and ≥71.0kg were significantly associated with successful treatment outcome [41], this might be explained by under nutrition which increases the risk of advanced TB disease and lowers immune response, resulting to poor treatment outcome. This study revealed that TB/HIV co-infection was associated with poor treatment outcome. HIV negative TB patients had higher odds of successful treatment outcome compared to HIV positive TB patients. This is in contrast to finding among persons in prison in northern Ethiopia where HIV co-infection was not associated with treatment outcomes but similar to that in Ethiopian university hospital and persons in prison in Zomba Malawi [27, 28, 41]. This could be attributed to the immune compromised associated with both disease, poor adherence to drugs due to large daily pills intake and the negative drug-to-drug interaction between Anti-TB and Anti-Retroviral drugs.

Although to the best of our knowledge, this study is the first among the studied population but it is limited by our inability to add other variables of interest which were not in the TB treatment register and may influence the treatment outcome such as height so we couldn’t calculate Body Mass Index (BMI), educational status, cigarette smoking, comorbidity, substance use as the study relied on historical records. Also data analysis from historical records might affect the validity of the results and the study used 5.4% of the national inmates’ population which might affect the generalizability of the study. For the purposes of understanding the associated factors for treatment outcome of drug-susceptible TB among inmates in prison settings in Bauchi State, the sample was adequate.

## Conclusion

This study demonstrated that TB treatment success rate among inmates in prison settings in Bauchi state was lower than the recommended WHO target and the national average. The TSR in this study was significantly influenced by age, duration of imprisonment, pretreatment body weight and HIV status, hence to improve TB TSR among persons in prison, these factors should be the major consideration during drugs adherence, psychological and nutritional counselling and the Bauchi state correctional service authority should develop a follow-up strategy to track inmates with TB transferred-out or discharged from prison while on treatment, this has impacted negatively on the treatment outcome and has implication for TB control programme with the risk of those who interrupted treatment developing drug-resistant TB and could serve as reservoir for the transmission of resistant strains to contacts. Dissemination meeting was held with the TB desk officers and the superintendents of the five prison settings where findings were communicated for sensitization and resolutions reached for improved treatment outcome.

## Supporting information

S1 Dataset (XLSX)

## Abbreviation

DOTs: Directly Observed Treatment short-course
EPTB: Extra Pulmonary Tuberculosis
HCW: Healthcare Worker
HIV: Human Immunodeficiency Virus
MDR: Multi-Drug Resistant
SNPTB: Smear Negative Pulmonary Tuberculosis
SPPTB: Smear Positive Pulmonary Tuberculosis
TB: Tuberculosis
TSR: Treatment Success Rate
